# Autoimmune Hemolytic Anemia as Presenting Symptom of Hodgkin Lymphoma

**DOI:** 10.1155/2021/5580823

**Published:** 2021-03-31

**Authors:** Nicholas B. Burley, Paul S. Dy, Suraj Hande, Shreyas Kalantri, Chirayu Mohindroo, Kenneth Miller

**Affiliations:** ^1^Department of Internal Medicine, Sinai Hospital of Baltimore, Baltimore, MD, USA; ^2^Department of Hematology/Oncology, Sinai Hospital of Baltimore, Baltimore, MD, USA

## Abstract

Autoimmune hemolytic anemia (AIHA) is related to an underlying condition in an estimated 50 to 60%, while the remaining is idiopathic, as a result of a combination of immune activation, deficiency, or dysregulation. AIHA is associated with viral infections, autoimmune disorders, immunodeficiencies, lymphoproliferative disorders, and pregnancy. AIHA has predictive properties and may be a harbinger of future lymphoproliferative disorders in up to 20% of AIHA cases. Autoimmune hemolytic anemia (AIHA) has been associated with lymphoproliferative disorders particularly chronic lymphocytic leukemia and non-Hodgkin lymphoma. Rarely is it seen in Hodgkin disease. In the following report, we describe the presentation of AIHA, ultimately resulting in the diagnosis of nodular sclerosis Hodgkin lymphoma (stage III). From the limited reports and reviews available, it is understood that advanced Hodgkin (stage III or IV) of nodular sclerosis (NS) or mixed cellularity (MC) types portend a stronger affiliation to AIHA. The majority of AIHA-associated Hodgkin lymphoma presents as stage III or IV disease with the hemolysis being the presenting symptom, as in this case. The mainstay of AIHA therapy has been corticosteroids; however, this first-line regimen appears to be less effective when treating AIHA in the setting of HL. The exact mechanism of AIHA related to HL is unclear, and it may be thought to be that tumor cell produced autoantibodies. Other hypotheses include paraneoplastic phenomena or more, perhaps immunity to tumor cells may cross-react with antigens on the red cells. Although these mechanisms require further investigation, the relationship of the AIHA and HL represents a piece to a larger puzzle between autoimmune disorders and lymphoproliferative conditions.

## 1. Introduction

The etiology of autoimmune hemolytic anemia (AIHA) is related to an underlying condition in an estimated 50 to 60%, while the remaining is idiopathic, as a result of a combination of immune activation, deficiency, or dysregulation [1]. AIHA is associated with viral infections, autoimmune disorders, immunodeficiencies, lymphoproliferative disorders, and pregnancy. This group of AIHA disorders can be related to autoimmune lymphoproliferative syndrome (ALPS), chronic lymphocytic leukemia (CLL), lymphoma, and clot agglutinin-related disease. AIHA has predictive properties and may be a harbinger of future lymphoproliferative disorders in AIHA cases [2]. While AIHA has well-known associations with non-Hodgkin lymphoma, its relationship to Hodgkin lymphoma is rare and potentially less recognized. Limited reports and reviews suggest that NS, MC, and stages III and IV HL are more commonly associated with AIHA [3]. AIHA may be the presenting symptom leading to the diagnosis of Hodgkin lymphoma (HL). In the following report, we describe the presentation of AIHA ultimately resulting in the diagnosis of nodular sclerosis Hodgkin lymphoma (stage III).

### 1.1. Case Summary

A 20-year-old female college student with no medical comorbidities presented to the hospital with progressive presyncopal symptoms, exertional dyspnea, and fatigue that was worsening over the course of several months. She noted increased difficulty with exertion, particularly climbing several flights of stairs requiring several breaks to recover. At the time of presentation, she had worsened presyncope, diaphoresis, and severe generalized weakness. She reported no recent illness, travel, sick contacts, or high-risk behavior (i.e., sexual activity and intravenous drug use), except for occasional nonbinge drinking alcohol use.

On initial examination, she was found to have conjunctival pallor, left supraclavicular lymphadenopathy (measured 3-4 cm width), lower anterior cervical firm adenopathy (measured 3-4 cm width), left axillary fullness without discrete masses, and a palpable spleen tip below the costal margin.

Preliminary laboratory results revealed white blood cell count 1.73 K/mm^3^, hemoglobin 4.6 mg/dL, hematocrit 15.7%, reticulocyte 9.9%, haptoglobin <20 mg/dL, total bilirubin 1.3 mg/dL, direct bilirubin 0.30 mg/dL, and lactate dehydrogenase 312 unit/L. White blood cell differentiation revealed reduced absolute lymphocyte count of 0.74 K/mm^3^ although relatively increased differential of 44%. Additional laboratory studies included a positive direct Coombs test for warm A plasma, IgG and C3, and cold agglutinin positivity. Further autoimmune antibody screening tests were not pursued. Her erythropoietin level was elevated erythropoietin 579.1 milli units/ml. Folate and B12 levels were within normal limits. Free light chains kappa and lambda showed no monoclonal gammopathy. Serum protein electrophoresis showed no evidence of monoclonal protein in the gamma region. Urine protein electrophoresis was negative for monoclonal protein in the gamma region.

**Figure 1:**
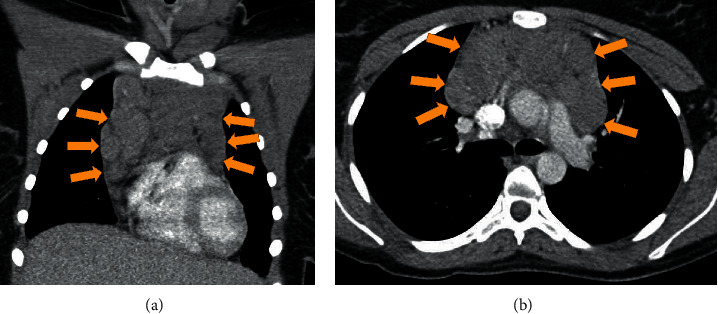
CT thorax demonstrating multiple pathologically enlarged left supraclavicular lymph nodes associated with enlarged partially visualized left cervical lymph nodes. Mass effect on the left lobe of the thyroid gland and the trachea from the pathologically enlarged left supraclavicular lymph nodes. Pathologically enlarged lymph nodes throughout the anterior mediastinum. Enlarged right paratracheal lymph nodes as well. Pathologically enlarged left axillary and subpectoral lymph nodes as well.

Computed tomography of thorax, abdomen, and pelvis demonstrated diffuse bulky adenopathy (see Figure 1), as well as splenomegaly and retroperitoneal adenopathy.

A core needle biopsy of the supraclavicular lymph node was performed, and pathologic evaluation demonstrated classical Hodgkin lymphoma with Reed–Sternberg cells expressing CD15 and CD30. Evaluation of the bone marrow demonstrated no evidence of Hodgkin lymphoma. After further imaging and biopsies, the patient was diagnosed with stage III classic Hodgkin lymphoma (nodular sclerosing) after the presenting symptom of warm autoimmune hemolytic anemia.

This patient's anemia was highly symptomatic. Multiple units of red cells were ordered but were delayed due to her multiple antibodies. After receiving blood, her symptoms significantly improved with repeat hemoglobin greater than 9.0 mg/dL. Patient's hemolysis was managed with intravenous methylprednisolone 1 mg/kg every 6 hours during her hospital stay lasting several days which was then tapered to oral prednisone 40 mg daily until the initiation of her chemotherapy regimen of Adriamycin, brentuximab-vedotin, vinblastine, and dacarbazine (A(BV)VD).

In the oncology clinic, after completing two cycles with A(BV)VD of a planned six cycles, the PET scan showed good response to treatment with a marked decrease in size and FDG uptake in the left neck and mediastinal nodes SUV max of 1.58 compared to pretreatment SUV max of 15.6. There were residual nodes in these regions having very low-level FDG activity equal to that of the mediastinal blood pool. No new site of disease was seen. With chemotherapy patient's hemolysis resolved, steroids were tapered and discontinued. Further re-evaluation of plasma IgG, C3, and cold agglutinin statuses were not pursued after chemotherapy given patient's excellent recovery.

At this time, the patient has completed six cycles of A(BV)VD with complete metabolic resolution and PET avidity resolution and is not recommended for radiation therapy. She is planned for ongoing surveillance.

## 2. Discussion

The majority of AIHA is related to an underlying etiology that alters immune system activity such infectious, autoimmune disorders, immunodeficiencies, lymphoproliferative disorders, and pregnancy. The lymphoproliferative disorders are comprised of diseases such as ALPS, CLL, non-Hodgkin lymphoma, and Hodgkin lymphoma [3–6]. While AIHA has well-known associations with non-Hodgkin lymphoma and CLL, its relationship to Hodgkin lymphoma is rarer and potentially less well recognized. In one German study, the incidence of AIHA in Hodgkin disease is 0.2% [7].

From the limited reviews, it is understood that advanced Hodgkin (stage III or IV) of nodular sclerosis (NS) or mixed cellularity (MC) types portend a stronger affiliation to AIHA [8, 9]. In these limited studies, some demographic associations have been identified including advanced age, male sex, and laboratory findings such as high reticulocyte count, high ESR, and low absolute lymphocyte count [10]. The most identified HL subtypes were nodular sclerosing and mixed cellularity subtypes [11]. Notably, there are been cases that indicate that tumor burden may not necessarily relate to the onset of AIHA [12]

In the limited reports of AIHA in HL, there is a potential for worsened prognostication although this may coincide with the typically late-presenting stage of the disease rather than the AIHA itself [13].

While anemia is commonly present in HL, approximately a third of cases, which is typically related to chronic inflammation, further investigation for other etiologies should be considered, especially in the setting of hemolysis.

The exact mechanism of AIHA related to HL is unclear, and it may be thought to be that tumor cell produced autoantibodies as the majority are direct antibodies testing positive [7]. Other hypotheses include paraneoplastic pathways or autoimmunity of antibodies targeting the tumor cells that also affect the red blood cells. Although these mechanisms require further investigation, the relationship of the AIHA and HL represents a piece to a larger puzzle between autoimmune disorders and lymphoproliferative conditions [3, 6, 14].

In very rare cases, HL-related AIHA may be direct antiglobulin test negative which may be attributed to undetectably low levels of IgG, low-affinity bound IgG antibodies are washed away during pretest processing, or non-IgG antibodies are the cause of the AIHA (e.g., IgA and IgM) [15].

The mainstay of AIHA has been corticosteroids; however, this first-line regimen appears to be less effective when treating AIHA in the setting of HL [10, 16]. In one single-center study, the use of rituximab, traditionally a second-line therapy, had significantly improved mortality rates compared to corticosteroids in those with warm AIHA and lymphoproliferative disease [16]. As seen in this case, treatment of the primary disease with combination chemotherapy led to treatment of AIHA. In very rare cases, splenectomy may be advocated as an effective therapy [17].

While there is much to be elucidated about the relationship between AIHA and HL, it is clear that further study must be done into the interactions of lymphoproliferative diseases, their effects on the immune system, and the development of autoimmune disorders. As we continue to develop foundational knowledge of these interactions, greater understanding of best treatments for AIHA in the setting of HL will aid in management of this complication and potentially reveal further mechanisms to research.
